# Identification and genetic characterization of Jingmen tick virus from ticks sampled in select regions of Kenya; 2022–2024

**DOI:** 10.1371/journal.pone.0329878

**Published:** 2025-10-13

**Authors:** Lydia Mwasi, Samoel Khamadi, Wallace Bulimo, Johnson Kinyua, Santos Yalwala, Luis Pow Sang, Haynes Robert, Gerald G. Kellar, John Eads, Fredrick Eyase

**Affiliations:** 1 Kenya Medical Research Institute, Center for Global Health Research, Kisumu, Kenya; 2 Jomo Kenyatta University of Agriculture and Technology (JKUAT), Nairobi, Kenya; 3 Kenya Medical Research Institute, Centre for Virus Research, Nairobi, Kenya; 4 Walter Reed Army Institute of Research-Africa/Kenya Medical Research Institute, Kisumu, Kenya; 5 Walter Reed Army Institute of Research-Africa, Nairobi, Kenya; Beni Suef University Faculty of Veterinary Medicine, EGYPT

## Abstract

Jingmen tick virus (JMTV), an emerging segmented RNA virus classified as an ungrouped flavivirus, poses a growing public health concern globally. Known for its association with febrile illnesses and wide host range, JMTV has been detected in Rhipicephalus, Hyalomma, and Amblyomma ticks collected from cattle, goats, sheep, camels, and chickens in pastoral regions of Kenya, including Baringo, Mandera, Malindi, Lamu, Mombasa, Wajir, Isiolo, and West Pokot. Using viral metagenomics next-generation sequencing, this study analysed adult ticks (n = 1629, 72 pools). A total of 53% (38/72) pools were positive for at least one viral pathogen, with JMTV detected in 87% (33/38) of these pools across all study sites. Phylogenetic analyses revealed evidence of distinct Kenyan JMTV strains, with sequence segments from Malindi and Wajir clustering uniquely in their own clade; suggesting potential localised evolutionary pressures. Time calibrated phylogeny for the segment 1(RdRp) suggested varied ancestral origins and evolutionary relationships for the JMTV strains. MEME, BUSTED and FUBAR methods implemented in the Data-Monkey, unanimously identified codon 290 in segment 1 and 30 in segment 4 to be undergoing episodic positive selection. Recombination analysis performed using the RDP4 recombination detection tool indicated a recombination event in segment 2 of the Lamu JMTV strain that was confirmed by seven detection methods of the RDP4 tool and visualised in BootScan. These findings suggest that Kenyan JMTV strains are undergoing positive selection, potentially driven by unique ecological and host factors. Segmented genome evidence of recombination highlights the increasing virus’s potential for antigenic diversity. Host diversity and virus phylogenetic patterns underscore the zoonotic potential and its capacity for regional spread, emphasizing the critical need for enhanced vector surveillance. Temporal and ecological drivers like seasonal tick activity and livestock movement warrant investigation to elucidate JMTV transmission dynamics. Prioritizing tick-borne virus surveillance in Kenya will strengthen public health strategies and mitigates emerging viral risks.

## Introduction

Emerging and re-emerging pathogens are a great public health concern due to their potential to cause outbreaks [1]. Jingmen viruses have recently been identified and taxonomically described as unclassified flaviviruses [1,2]. The first Jingmen virus was isolated in China from a *Rhipicephalus microplus* tick in 2010 [2]. The virus has two phylogenetic clades; one clade covers viruses from ticks, mosquitoes, humans, cattle, monkeys, bats, rodents, sheep, and tortoises and has been observed to cause febrile illness and flu-like symptoms in humans [2]. The second clade has been isolated from insect species, crustaceans, plants, and fungi and has no confirmed cases of human infections [2]. Jingmen tick virus (JMTV) has a wide host range and viral transmission among ticks is efficient through “cofeeding”; it is termed as a true arbovirus, with a cycle involving an arthropod vector and a vertebrate host [3]. The fact that the virus has a wide host range and is distributed across different regions, makes it an emerging pandemic capable virus.

Unlike flaviviruses, the Jingmen virus is segmented with 4–5 segments; segment 1 of the Jingmen virus encodes for Non-Structural Protein 1 (NSP1), and resembles the flavivirus NS5-like protein that includes an RNA-dependent RNA polymerase (RdRp) and methyltransferase [4]. Segment 2 encodes for Viral Protein 1(VP1); Segment 3 encodes for Non- Structural Protein 2 (NSP2), which also shares similarities with the NS2B/NS3 flavivirus protein complex that encompasses transmembrane regions, serine protease and helicase domains, while Segment 4 encodes the Viral Protein 2–3 (VP2–3) [4]. Segments 2 and 4 are suggested to be structural proteins that constitute the envelope, capsid, and membrane proteins, respectively [4]. In addition, an open reading frame (ORF) overlapping with VP1 coding sequence at the beginning of segment 2 (VP4) is documented to encode for a small membrane protein [5]. The possible ancestral origin of non-structural proteins of segment 1 (NSP1) and segment 3 (NSP2) of the JMTV from flaviviruses suggest a unique evolutionary link between segmented Jingmen viruses and the non-segmented flaviviruses [6]. The overall length of the JMTV genome is 11,401 nucleotides, similar to that of flaviviruses [4].

In Kenya, JMTV has previously been isolated from Rhipicephalus, Hyalomma and Amblyomma tick species from goats, sheep, tortoise and cattle in Kajiado and Baringo pastoral regions [6]. Although there is evidence of circulation of the virus in these two pastoral regions in Kenya, there is no surveillance data of the virus in other pastoral regions of Kenya. This study addresses this gap by conducting a cross sectional study across select pastoral regions including Baringo, Mandera, Kilifi, Lamu, Mombasa, Wajir, Isiolo, and West Pokot counties. Baringo county previously reported Jingmen virus circulation [6], the Mandera county borders Ethiopia to the North, Wajir county borders Somalia to the West, Lamu county borders Somalia to the North and West Pokot county borders Uganda to the East; this trends towards surveilling for possible cross-border importation of vectors and consequently pathogen transmission due to transboundary movement of livestock. The pastoral nature of the study counties offers valuable insights into the prevalence and diversity of Jingmen tick virus in Kenya, highlighting its spread across the country and possible transmission to humans.

Unbiased metagenomic next generation sequencing (mUNGS) enables the identification of total microbiomes, making the identification of novel and emerging pathogens possible. In the present study Jingmen tick virus was identified and characterized, using this method.

## Materials and methods

### Ethical approval

The study was approved by the Kenya Medical Research Institute (KEMRI) Scientific and Ethics Review Unit (SERU) under protocol number KEMRI/SERU/CVR/4845 and license number 32115 by the National Commission for Science, Technology & Innovation (NACOSTI). The study was also submitted for approval to the Walter Reed Army Institute of Research (WRAIR) Institutional Review Board (IRB) under package WRAIR # 3192.

### Sampling and pooling

**Fig 1:**
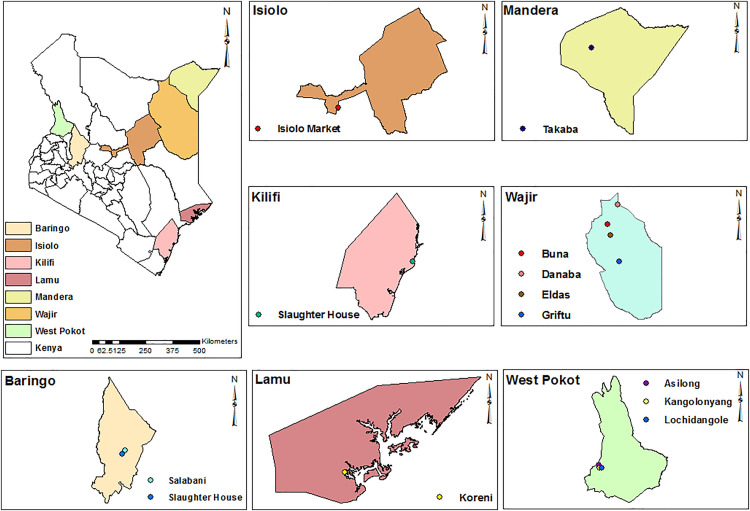
Tick sampling sites. This map was generated using ArcGIS version 10.2.2 for Desktop. Base maps, boundaries and shape files of Kenyan map and administrative boundaries of the Counties were derived from GADM data version 4.1 (https://gadm.org) and the maps were generated using ArcGIS Version 10.2.2 (http://desktop.arcgis.com/en/arcmap) advanced license) courtesy of Samuel Owaka.

Ticks were collected by grooming from cattle, sheep, goats, and camels from various pastoral counties including West Pokot (Lochidangole, Kangolonyang, Asilong), Isiolo (Market), Baringo (Salabani, Slaughter house), Mandera (Takaba), Lamu (Koreni), Wajir (Griftu, Danaba, Eldas, Buna) and Kilifi (Malindi, Slaughter house) (Fig 1). In Lamu, sample collection was carried out during the month of October in Lamu, with 37 mm precipitation and temperatures of 86–88°F. In Mandera, collection was done in June where temperatures of 67.5–84°F, precipitation of 1 mm and humidity of 50% were experienced. In Isiolo, collection was done in March with temperatures of 74–84°F and precipitation of 43 mm. In Kilifi, collection was done in November with temperatures of 73.4–87.8°F, precipitation of 112.2 mm and humidity of 81%. In Baringo, collection was done in November, with temperatures of 75–86°F and 58 mm of precipitation. In West Pokot, collection was done in March, with temperatures of 54–79°F, precipitation of 67.5 mm and humidity of 61%. Lastly in Wajir, collection was done in February, with temperatures of 75–97°F, precipitation of 2.1 mm and humidity of 53%. The collected ticks were placed in 15 mL centrifuge tubes (Corning Inc, Corning, NY, USA) and transported to the KEMRI/ Walter Reed Army Institute of Research-Africa (WRAIR-A) laboratories on dry ice. All the ticks collected (fed, engorged and unfed ticks) were morphologically identified using taxonomical keys [7] and pooled irrespective of their feeding status and animal host, into groups of 1–8 ticks. Pooling of ticks from different domestic animals was randomly performed with respect to their species and site of collection and stored at −81°C prior to laboratory methods [6].

### Tick homogenization

Frozen individual pools of ≤10 ticks were homogenized with 0.7 ml dry volume 2.0 mm Zymo Bashing Beads (ZR S6003) in a mini-beadbeater-24 (Fisherbrand™) for 1 minute, with a mid-way pause of 10 seconds. One mL homogenizing media containing minimum essential medium, with Earle’s salts and reduced sodium hydrogen carbonate (NaHCO3), 15% fetal bovine serum (Gibco), 2% L-glutamine (Sigma Aldrich) and antimycotic/antibiotic solution (Sigma Aldrich) was added. The homogenates were vortexed and clarified through 10 minutes of 10,000 X g centrifugation (Eppendorf 5430-R) and filtered through a 0.22µm syringe filter (MilliporeSigma™, USA) to remove tissue debris and reduce quantities of bacteria and other impurities [8,9]. The homogenates were stored at −80°C prior to RNA extraction and metagenomics.

### RNA extraction and metagenomics

Total RNA was extracted from 100 µL of tick homogenates using the Zymo Quick-RNA™ Kit (USA), following the manufacturer’s instructions that included a DNase digestion step to digest free nucleic acid. The remaining unfiltered homogenates were stored at −80°C for viral isolation.

A sequencing library was prepared using the Illumina Ribo-Zero Plus Ligation kit (Illumina, USA) following the manufacturer’s instructions. RNA was quantified on a Qubit machine before library preparation using the RNA High Sensitivity (HS) kit (Invitrogen™) following ribosomal RNA depletion and DNA High Sensitivity (HS) kit (Invitrogen™) following cDNA synthesis. The 2100 Bioanalyzer (Agilent™) was used to quantify the final library before pooling and loading on the Illumina Miseq platform for sequencing with 300 *2 cycles.

### Sequence analysis

Sequence analysis was performed in Terra.bio [10] using the Theiameta_illumina_Pe_version PHB 2.1.0 workflow, where read quality trimming and adapter trimming was performed using the Trimmomatic tool v0.39 [11]. Read quantification was performed using fastq-Scan v0.4.4 [12], and taxonomic classification was performed in Kraken2 v2.1.2 [13]. De novo metagenomic assembly was performed using metaSPAdes v3.12.0 [14]; assembly alignment and contig filtering was done by SAMtools v1.17 [15] and Minimap2 v2.22-r1101 [16] using a reference genome from NCBI. Comprehensive microbial variant detection and genome assembly improvement was performed by in Pilon v1.24 [17] and assembly quality assessment performed by the quality assessment Tool (QUAST) v5.0.2 [18] to give FASTA format contigs.

### Phylogenetic analyses

Maximum likelihood (ML) phylogenetic trees were estimated using Jones-Taylor-Thornton (JTT) model based on comparison of translated amino acid sequences of the four JMTV segments in MEGA 7 v7.0.26 with 1000 bootstrap replications nodal support [19,20], viewed using Figtree v1.4.4 [21] and midpoint rooted. Additionally, a phylogenetic time calibrated tree was generated for the study segment 1 genomes in BEAST v1.10. Briefly, 85 JMTV sequences were obtained from GenBank by country of origin and year of sample collection for inclusion in the calibrated tree analysis. Sequences were aligned using MAFFT software v7.310 and editing was performed in BioEdit software v7.7.1. BEAST was used to estimate the evolutionary rate in segment 1 (RdRp) by the relaxed uncorrelated lognormal molecular clock model. The maximum clade credibility (MCC) tree was run with a chain length of 250,000,000 replications; confirmation of convergence of all parameters in Tracer v1.7.2 (effective sampling size (ESS)> 200). The final MCC tree was generated by TreeAnnotator with a Burn-in of 10% of the total chain length and the tree was visualized by Figtree v1.4.4.

### Natural selection and recombination analyses

A mixed-effects maximum likelihood approach for individual sites under episodic positive selection was performed using the Mixed Effects Model of Evolution (MEME) analysis in Data monkey [22,23]. The results were confirmed using the Fast, Unconstrained Bayesian AppRoximation (FUBAR) [24] and the Branch-Site Unrestricted Statistical Test for Episodic Diversification (BUSTED) approaches in Data monkey [25].

Recombination analysis was performed using seven methods RDP, GENECONV, BOOTSCAN, MaxChi, Chimaera, SiScan and 3Seq implemented in RDP4 to identify and confirm potential recombination [26,27]. At least four of the seven methods had to detect recombination to consider a recombination event as significant and accurate; p-value cut off of 0.05 [28]. SimPlotv3.5.1 was used to generate recombination plot maps for visualisation and validation of recombination events detected by RDP4 [29].

## Results

### Tick collection and morphological identification

A total of 1,629 ticks were collected from domestic animals in the study counties as follows: Isiolo (Market n = 334); Lamu (Koreni n = 25); Kilifi (Malindi, Slaughter house n = 138); Mandera(Takaba n = 147); Baringo (Salabani n = 300,Slaughter house n = 116); Wajir (Griftu n = 102,Danaba n = 39,Eldas n = 43 and Buna n = 2) and West Pokot (Lochidangole n = 48,Kangolonyang n = 9, Asilong n = 88) (Table 1).The ticks collected in this study from different domestic animals, were both hard and soft ticks belonging to 4 genera and 14 species (Table 1): *Amblyomma gemma* (13.5%), *Amblyomma lepidium*(6.3%), *Amblyomma variegatum*(7.7%), *Argas persicus* (18.4%), *Hyalomma albiparmatum* (2.3%), *Hyalomma anatolicum anatolicum* (0.4%),*Hyalomma dromedarii* (4.1%), *Hyalomma impeltatum* (0.4%), *Hyalomma marginatum rufipes*(17.4%), *Hyalomma truncatum* (7.9%), *Rhipicephalus appendiculatus* (4.2%), *Rhipicephalus boophilus microplus* (9.8%), *Rhipicephalus evertsi evertsi* (3.3%), and *Rhipicephalus pulchellus* (4.4%).

**Table 1:** Number of ticks collected across study sites.

	Lamu	Isiolo	Kilifi	Wajir	Mandera	West Pokot	Baringo	Total Per Species
*Amblyomma gemma*	8	133	48		31			220
*Amblyomma lepidium*		12	16	36	1	37		102
*Amblyomma variegatum*		1	4		87	34		126
*Argas persicus*							300	300
*Hyalomma albiparmatum*			24	7	6			37
*Hyalomma anatolicum anatolicum*				6				6
*Hyalomma dromedarii*			1	42	23			66
*Hyalomma impeltatum*				6	1			7
*Hyalomma marginatum rufipes*	9	97	47	80	51			284
*Hyalomma truncatum*		11	1			19	98	129
*Rhipicephalus appendiculatus*		2	31		1	34		68
*Rhipicephalus boophilus microplus*	6	33	86			16	18	159
*Rhipicephalus evertsi evertsi*			25		23	5		53
*Rhipicephalus pulchellus*	2	45	8	9	8			72
Total Per Site	25	334	291	186	232	145	416	1629

### Prevalence and percent homology of JMTV sequences

The ticks were pooled ≤10 based on tick species, animal host and collection site resulting in 231 pools [29]. Aliquots from the original pools were further pooled into 72 super pools of ≤50 per tick species and site of collection. A total of (38/72) 53% of pools were positive for at least one viral pathogen, with JMTV detected in 87% (33/38) of these pools across all study sites. The Jingmen tick virus segment sequences were deposited in GenBank under the accession numbers PV384450 - PV384535 under BioProject ID: PRJNA1238498. Sequences included in this manuscript (S1 Table).

The study JMTV sequences had a nucleotide homology of 89–94% with JMTV sequences from Kenya, Uganda, China, and Japan, and amino acid homology of 90–99% with JMTV sequences from Kenya, Uganda, China, Laos, and Japan (S2 Table).

### Phylogenetic analyses

**Fig 2:**
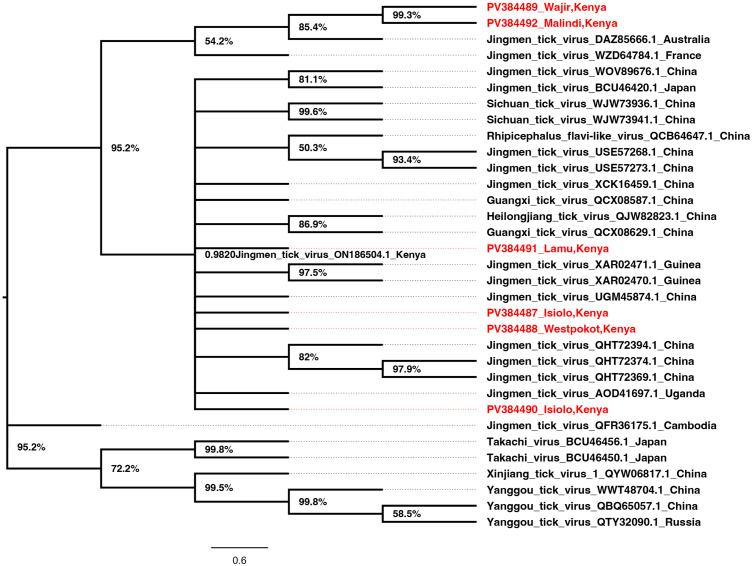
Phylogenetic tree for JMTV segment 1 (NSP1). Amino acid maximum likelihood tree performed in MEGA 7 version 7.0.26 with 1000 bootstrap replications. Tree midpoint rooted and viewed using Figtree version 1.4.4.

**Fig 3:**
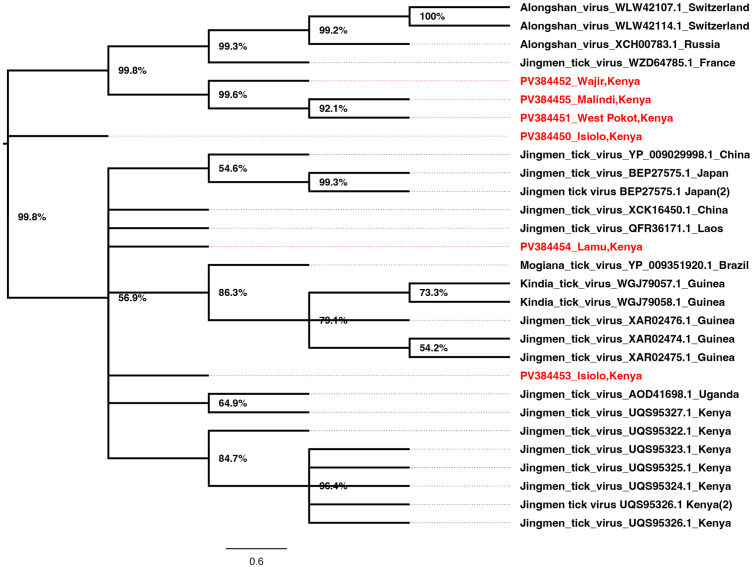
Phylogenetic tree for JMTV segment 2 (VP1). Amino acid maximum likelihood tree performed in MEGA 7 version 7.0.26 with 1000 bootstrap replications. Tree midpoint rooted and viewed using Figtree version 1.4.4.

**Fig 4:**
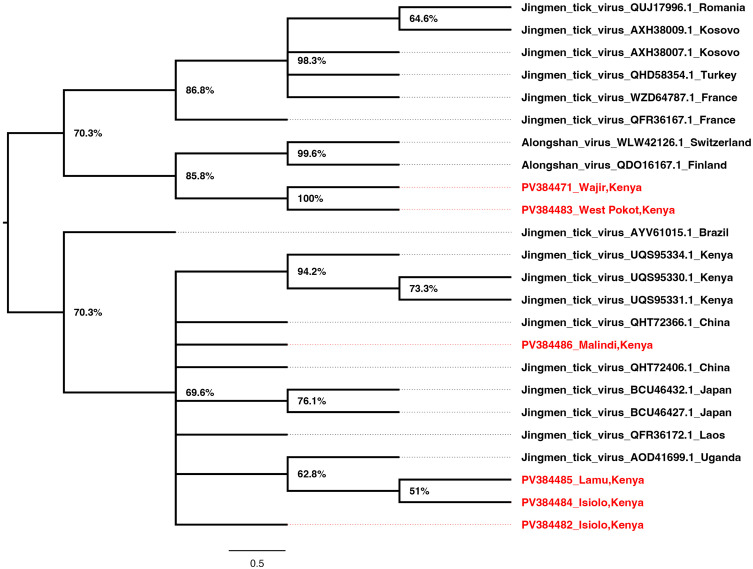
Phylogenetic tree for JMTV segment 3 (NSP2). Amino acid maximum likelihood tree performed in MEGA 7 version 7.0.26 with 1000 bootstrap replications. Tree midpoint rooted and viewed using Figtree version 1.4.4.

**Fig 5:**
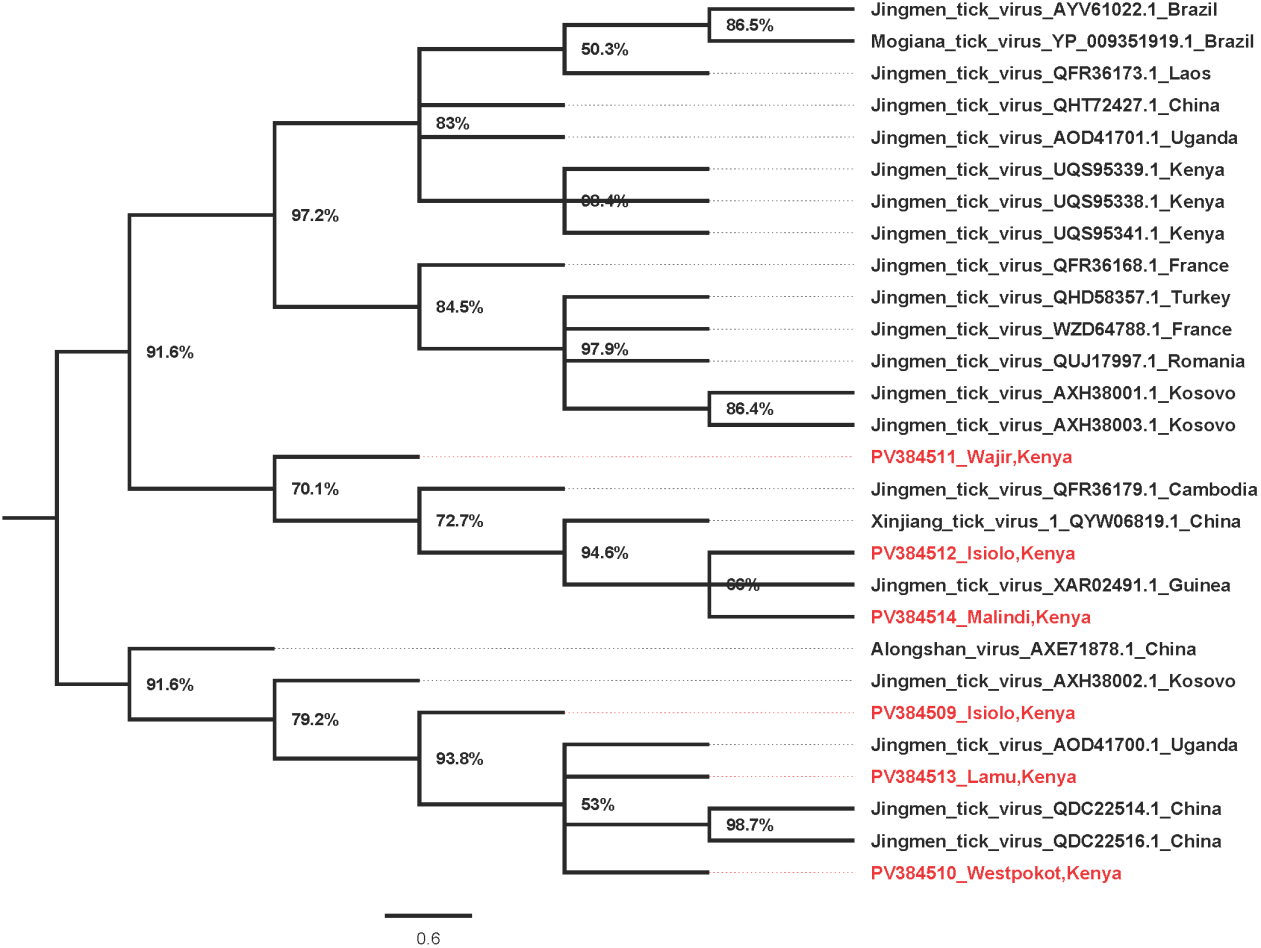
Phylogenetic tree for JMTV segment 4 (VP2&3). Amino acid maximum likelihood tree performed in MEGA 7 version 7.0.26 with 1000 bootstrap replications. Tree midpoint rooted and viewed using Figtree v 1.4.4.

JMTV Segment 1 for sequences from Kilifi/Malindi and Wajir clustered together in a well-supported and distinct clade. Those from Lamu, Isiolo, and West Pokot clustered in a second clade together with other strains from around the world (Fig 2). Interestingly, JMTV segment two sequences from Wajir, Kilifi/Malindi, and West Pokot clustered in a similar clade as segment 1. The strains from Russia, France, and Switzerland clustered in a second clade while sequences from Lamu and Isiolo clustered in a third clade with the rest of the strains from around the world (Fig 3). The JMTV segment 3 sequences from West Pokot and Wajir clustered in one clade maintaining the tree topology similar to segment 1 and segment 2 trees. The segment 3 sequences from Isiolo, Kilifi/Malindi, and Lamu clustered with the rest of the strains from around the world (Fig 4). Finally, for segment 4 JMTV sequences, Wajir, Kilifi/Malindi, and Isiolo clustered in one clade, with Cambodia, Guinea, and China. However, the sequences from Wajir and Cambodia formed distinct branches within the clade. Sequences from West Pokot and Lamu and a second sequence from Isiolo clustered in a second clade with the rest of the strains from around the world clustering together in a third clade (Fig 5).

**Fig 6:**
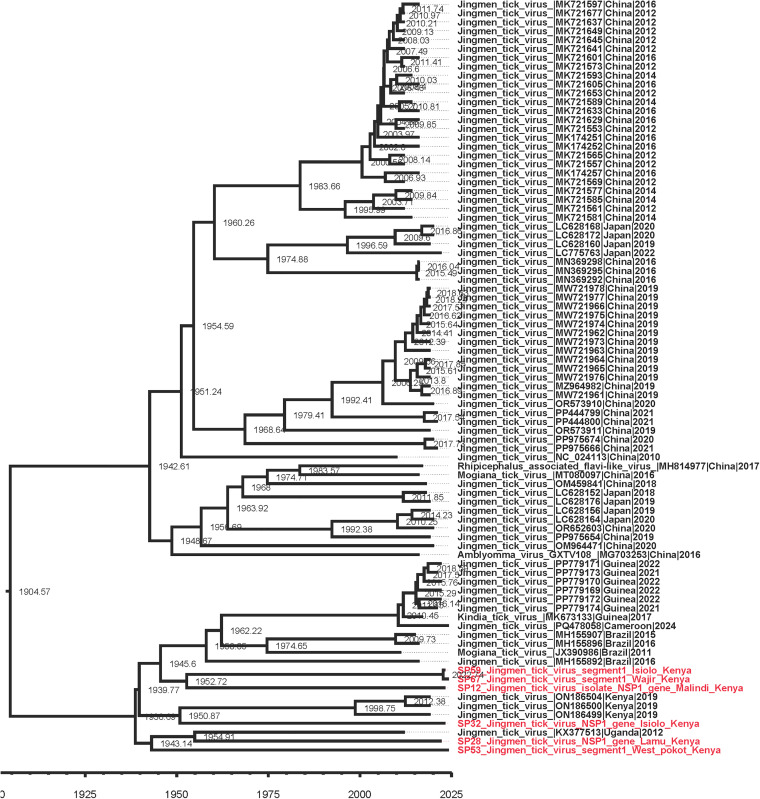
Time calibrated tree showing timescale of JMTV. A phylogenetic tree constructed based on segment 1 (RdRp) of JMTV.

**Fig 7:**
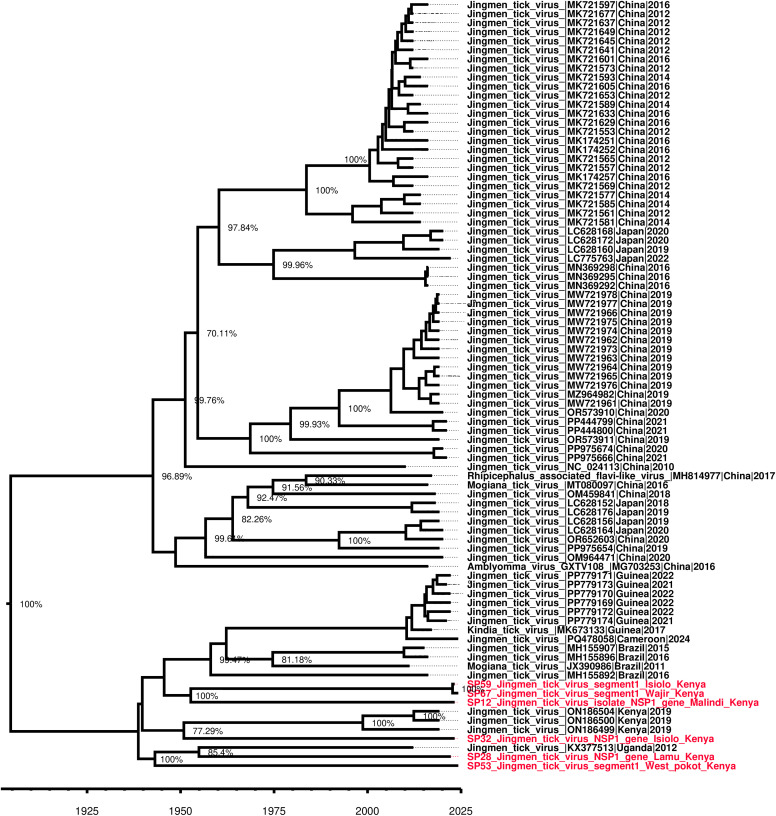
Time calibrated tree showing evolutionary relationships. A phylogenetic tree constructed based on segment 1 (RdRp) of JMTV.

The JMTV time calibrated tree showed one Isiolo strain branched from ancestral strain in 1950 while the second Isiolo strain, Wajir and Malindi/Kilifi strains branched from their ancestral strain in 2022. The Lamu strain branched from ancestral strain in 1954 while the West Pokot strain branched from ancestral strain in 1943. This suggests varied ancestral origins for JMTVs in the present study, and possible multiple introduction events (Fig 6). The data also shows one of the Isiolo strains, together with those from Wajir and Kilifi/Malindi forming a clade of their own; a second Isiolo strain formed a clade with other JMTV strains from Kenya while the Lamu strain formed a clade with JMTV strain from Uganda and in close proximity to the West Pokot strain (Fig 7). These findings suggest possible inter-country border transmission of JMTV to Kenya from Uganda through the border at West Pokot.

### Natural selection and recombination events

Episodic positive selection analysis using MEME, FUBAR, and BUSTED confirmed selection at codon site 290 in segment 1 and site 30 in segment 4 (Table 2).

**Table 2:** Codon sites under episodic positive selection pressure.

Virus	Segment	Codon	Methods		
			p-value		
			MEME	FUBAR	BUSTED
JMTV	Segment 1	290	0.025	0.035	0.000
	Segment 4	30	0.009	0.041	0.000

**Fig 8:**
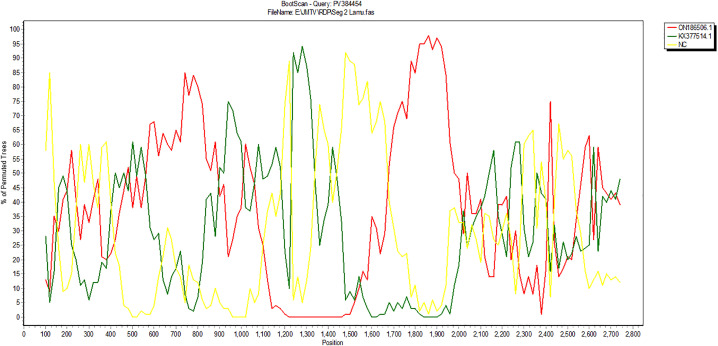
BootScan plot of segment 2 of JMTV genome for strain PV384454, with evidence of recombination. The plot was prepared within a sliding window of 200 bp, with a 20 bp step size, for 1000 replications (Gap Strip: On, Neighbor – joining, T/t:2.0).

We detected one recombination signal in JMTV segment 2 of the Lamu study strain, PV384454 that was confirmed by 7 of the 8 recombination tools in RDP4 (Table 3), with the Kenyan JMTV strain ON186506 as the parental sequence (S3 Table). The recombination observed in segment 2 is evidenced by BootScan recombination plot (Fig 8).

**Table 3:** Recombination signals detected in the JMTV segment 2 using tools in RDP4.

JMTV	NO	Parental Sequence	Breakpoint positions		Detection Methods (P value)						
			Begin	End	RDP	GENECONV	BOOTSCAN	MaxChi	Chimera	SiScan	3Seq
segment 2	PV384454	ON186506.1	6	124	1.129E-27	8.040E-50	–	7.751E-17	6.124E-10	4.492E-30	1.347E-04

## Discussion

Since its initial isolation in China in 2010, the JMTV has been detected widely across the globe in ticks, animal vertebrates, and humans [29,30]. JMTV was first identified from the *Rhipicephalus boophilus microplus* ticks in China in 2010 [1–4,6,28,31], however, over time, the virus has also been identified in other tick species [31–33], and in ticks from both wild and domestic animals, highlighting the wide vector and host adaptation of the JMTV virus [28]. RNA viruses are known to evolve through a complex interplay of long-term co-evolution with their hosts and repeated events of host switching [34–36]. Previous JMTV studies have suggested possible genetic reassortment and recombination in JMTV during cross-species transmission [28,36,37]. This rather complex ticks- vertebrates relationship has an impact on evolutionary relationships and consequently increases human infection risk [28]. The current study has obtained partial and near full-length sequences of JMTV strains from *Rhipicephalus boophilus microplus*, *Amblyomma variegatum, Hyalomma dromedarii* and *Amblyomma lepidium* ticks collected from Isiolo, West Pokot, Wajir, Kilifi/Malindi and Lamu regions of Kenya between 2022–2024. The overall positive- rate of JMTV in this study (45.8%) was comparatively higher than observed in a similar JMTV study in Turkey (3.9%) [33]. Comparative prevalence was observed in ticks from other studies in China (53–63%), Brazil (25–67%), Trinidad and Tobago (6–46%) and in the French Antilles (24–77%) [33–35]. In addition, our results are consistent with surveys conducted in all these studies; suggesting an increased risk of JMTV transmission among ticks through co-feeding in animals [28,33]. The geo-phylogenetic distribution of JMTV strains in the current study portrayed diversity in phylogenetic placement with other JMTV strains across the globe; suggesting possible movement between regions [32,36,37]. The phylogenetic analyses of JMTV genomic segments from different Kenyan counties reveal complex evolutionary dynamics that reflect both local diversification and global connectivity of tick-borne viral lineages. Segment 1 sequences from Kilifi/Malindi and Wajir that formed a distinct clade, suggest a degree of geographical structuring that may be linked to ecological or host-vector differences in these regions [38]. In contrast, segment 1 sequences from Lamu, Isiolo, and West Pokot clustered with international strains, implying historical or ongoing cross-regional viral exchanges. Such clustering with strains from multiple continents highlights the likelihood of viral introductions facilitated by livestock trade, bird migration, or movement of tick species across borders, consistent with previous observations in other tick-borne viruses [39–41].

The congruence of segment 2 topologies with segment 1, particularly for sequences from Wajir, Kilifi/Malindi, and West Pokot, further supports the stability of these local clades. However, the presence of separate groupings for Lamu and Isiolo suggests potential recombination or reassortment events, a phenomenon reported in JMTV and other multi-segmented tick viruses [42]. The detection of distinct clades involving strains from Europe and Africa underscores the global nature of JMTV diversity, reflecting both regional adaptation and transboundary viral dispersal [42].

Segment 3 phylogenies reinforced the pattern observed in segments 1 and 2, with Wajir and West Pokot sequences clustering together, while sequences from coastal and northern Kenya grouped with strains from Asia and Europe. This consistency across multiple segments suggests that some JMTV lineages are maintained locally, while others are influenced by global viral gene flow. The capacity of JMTV to adapt across ecological zones may reflect strong selective pressures at the wildlife–livestock–human interface, where ticks encounter diverse vertebrate hosts [31].

Segment 4 displayed more complex clustering, with Kenyan sequences grouping alongside strains from Cambodia, Guinea, and China. The branching patterns within this segment indicate both localized evolution, as seen with Wajir-specific branches, and integration into global lineages. Such heterogeneity may be driven by segment-specific evolutionary rates, consistent with evidence of genetic diversity and distinct sub-lineages in JMTV [43]. Studies have since suggested recombination and mosaic evolution in JMTV, highlighting its complex evolutionary dynamics [4,31].

The time-calibrated phylogenetic analysis provided insights into the historical emergence and diversification of JMTV lineages circulating in Kenya. The time calibrated trees for evolutionary relationships and time scale suggest multiple ancestry origins for the study strains and thus potential varied times of introduction into the country, further explaining the strain divergence across the study sites. The branching of the West Pokot strain to an ancestral origin in the 1940s, together with the Lamu strain’s divergence in the 1950s, points toward long-standing viral circulation in East Africa. The clustering of Lamu strains with Ugandan isolates highlights likely cross-border transmission, possibly mediated by livestock movement or vector dispersal across the porous Kenya–Uganda border. Such transboundary viral flow has previously been reported for other arboviruses, including Crimean–Congo hemorrhagic fever virus (CCHFV) and Rift Valley fever virus, where livestock trade and ecological continuity sustain cross-country transmission dynamics [44,45]. Livestock, such as cattle, sheep, and goats have been suggested to amplify CCHFV transmission by increasing tick populations, while wildlife acts as silent reservoirs, and birds may introduce infected ticks into new regions thus facilitating cross-border risk at the wildlife–livestock–human interface [44,45].

In contrast, the more recent divergence of Isiolo, Kilifi/Malindi, and Wajir strains in 2022 suggests either new introductions or recent expansions of viral populations. The existence of two genetically distinct Isiolo strains, one clustering with Wajir and Kilifi/Malindi, and the other aligning more closely with Kenyan JMTV lineages, demonstrates ongoing viral diversification. The observed heterogeneity is consistent with the broad genetic diversity documented among tick-borne viruses [39].

Positive episodic selection on segment 1 and 4 correlate with previous studies that have observed some evolutionary pressures in these JMTV segments [28], despite their conserved nature and important role in function of proteins they encode [28]. The segment 1 of JMTV encodes the non-structural protein NSP1, whose role is to mediate virus replication in the host while segment 4 encodes the viral protein 1 (VP1) and other proteins, related to changes in the virus’s ability to infect or transmit to other hosts. Evolutionary pressures at segments 1 and 4 suggests increased adaptability within the virus and its host for enhanced stability and dominance [34]. Beneficial mutations driven by positive selection often arise in RNA viruses under host pressures such as immune escape and adaptation, especially in functional regions like polymerase genes. This is supported by studies describing viral evolutionary mechanisms and host-virus co-evolution dynamics [46]. The presence of selective signals in JMTV suggests that viral replication machinery is responding to host or vector pressures, potentially facilitating cross-species spillover.

A recombination event observed on segment 2 of the Lamu strain PV384454 in comparison to previous strains from Kenya, China, and Uganda, with possible parental sequence to be that of a previous Kenyan strain ON186506 agree with previous studies that found JMTV segment 2 as having the fastest evolutionary rates in comparison to other JMTV segments [29]. The detection of recombination in segment 2 of the Lamu strain further emphasizes the dynamic evolutionary mechanisms acting on JMTV. Recombination is a pervasive evolutionary mechanism in RNA viruses, contributing to genetic diversity and enabling changes such as host range expansion and increased virulence [47]. In segmented RNA viruses such as influenza A, reassortment during co-infection is a fundamental driver of genetic diversity and emergence that has led to antigenic shifts that enabled host adaptation and pandemic spread in past outbreaks [48,49]. In JMTV, recombination has been reported in both Asian and European strains [42], and its presence in Kenyan isolates reinforces the notion that Africa may be a center for viral diversification. Recombination in JMTV has been documented during cross-species transmission, demonstrating its potential role in generating genetic diversity that may enhance ecological persistence [28].

## Conclusion

JMTV has been detected in ticks collected from domestic animals. The detection of viral pathogens in more than half of the tick pools (53%) indicates a substantial circulation of tick-borne viruses in the study area. The predominance of Jingmen tick virus (JMTV), which accounted for 87% of the positive pools (33/38), suggests that JMTV is the most widespread and dominant tick-borne virus in these populations. Its presence across all study sites highlights not only its broad geographical distribution but also its potential role as a key emerging pathogen of concern. The super pooling testing strategy had no effect on the sensitivity of the NGS assay because of the ability to detect viral pathogens in 53% of the tick pools; some of which had co-infections. This study has identified JMTV in 7 pastoral regions of Kenya, genetically characterizing the virus while demonstrating possible ancestral origin and timelines introduction to the country. Collectively, the phylogenetic, selection, and recombination evidence paints a picture of JMTV as a highly dynamic virus with multiple introduction events in Kenya, long-standing endemic lineages, and adaptive capacity to persist and diversify across hosts and regions. The Kenyan setting with its intersecting wildlife, livestock, and human populations, provides fertile ground for such evolutionary processes. These findings underscore the importance of sustained genomic surveillance, coupled with ecological and epidemiological monitoring, to anticipate the risk of JMTV emergence as a clinically significant pathogen. Future studies linking human and animal serological data would help to determine exposure to the virus. Studies to identify the growth potential and rate in relation to flaviviruses will further give more information about the nature of the Jingmen tick virus in Kenya.

## Supporting information

S1 Table Study JMTV strains and their GenBank Accession numbers. (PDF)

S2 Table JMTV nucleotide and amino acid percent homology with other global JMTV sequences. (PDF)

S3 Table JMTV recombination analysis sequences. (PDF)
