# Supplementary material for: Identification and genetic characterization of Jingmen tick virus from ticks sampled in select regions of Kenya; 2022–2024
Source: PLoS One. 2025 Oct 13;20(10):e0329878. doi: 10.1371/journal.pone.0329878 (PMC12517476; doi:10.1371/journal.pone.0329878)
Supplement: S1 Table — (PDF) [file pone.0329878.s001.pdf]

S1 Table. Study JMTV strains and their GenBank Accession numbers.

Sample Collection Site	Year of Collection	Tick Species	Segment 1	Segment 2	Segment 3	Segment 4
**Isiolo**	2023	*Rhipicephalus boophilus microplus*	PV384487(3079bp)	PV384450(2845bp)	PV384482(2812bp)	PV384509(2758bp)
**West pokot**	2024	*Amblyomma variegatum*	PV384488(3060bp)	PV384451(1418bp)	PV384483(1281bp)	PV384510(2740bp)
**Wajir**	2024	*Hyalomma dromedarii*	PV384489(1426bp)	PV384452(512bp)	PV384471(650bp)	PV384511(1343bp)
**Isiolo**	2023	*Amblyomma lepidium*	PV384490(3047bp)	PV384453(2775bp)	PV384484(2747bp)	PV384512(2726bp)
**Lamu**	2022	*Rhipicephalus boophilus microplus*	PV384491(3045bp)	PV384454(2776bp)	PV384485(2802bp)	PV384513(2814bp)
**Malindi**	2023	*Rhipicephalus boophilus microplus*	PV384492(2481bp)	PV384455(1447bp)	PV384486(2032bp)	PV384514(2579bp)
