# Supplementary material for: Identification and genetic characterization of Jingmen tick virus from ticks sampled in select regions of Kenya; 2022–2024
Source: PLoS One. 2025 Oct 13;20(10):e0329878. doi: 10.1371/journal.pone.0329878 (PMC12517476; doi:10.1371/journal.pone.0329878)
Supplement: S2 Table — (PDF) [file pone.0329878.s002.pdf]

S2 Table. JMTV nucleotide and amino acid percent homology with other global JMTV sequences.

Segment 1	nt % Homology	aa % Homology	Segment 2	nt %Homology	aa %Homology	Segment 3	nt %Homology	aa %Homology	Segment 4	nt %Homology	aa% Homology
**PV384487**	92.60% (LC628152)	97.7% (BCU46420)	PV384450	91.29% (LC628161)	94.37% (WWB03812)	PV384482	92.68% (MW722014)	98.82% (QFR36172)	PV384509	93.48% (KX377516)	97.03% (AOD41700)
**PV384488**	92.42% (MG703253)	98.39% (USE57273)	PV384451	91.48% (ON186507)	95.79% (WJW73937)	PV384483	94.15% (ON18514)	99.37% (UQ595332)	PV384510	93.34% (LC628155)	97.98% (AOD41700)
**PV384489**	92.15% (PP975670)	97.7% (QCB64647)	PV384452	89.45% (MH400990)	90.48% (QCB64648)	SP67_Wajir	93.85% (MH401002)	98.24% (AHZ31680)	PV384511	92.79% (MG703255)	98.57% (QHT72402)
**PV384490**	93.02% (LC628148)	98.74% (UYF11960)	PV384453	91.57% (PP975655)	96.06% (UQS95323)	PV384484	91.81% (MG703252)	98.69% (QFR36172)	PV384512	94.57% (KX377516)	100% (AOD41701)
**PV384491**	92.35% (LC628156)	98.74%(UYF 11960)	PV384454	91.26% (NC024112)	95.77% (UQS95323)	PV384485	91.61% (MG703252)	98.3% (BCU46437)	PV384513	94.18% (KX377516)	97.77% (AOD41700)
**PV384492**	92.1% (LC628164)	97.45%(QHT 72404)	PV384455	89.05% (OR03699)	94.8% (WWZ18059)	PV384486	89.36% (MW722014)	98.32% (UYF11978)	PV384514	91.08% (MK721572)	99.05% (WWZ18068)
