# Supplementary material for: Identification and genetic characterization of Jingmen tick virus from ticks sampled in select regions of Kenya; 2022–2024
Source: PLoS One. 2025 Oct 13;20(10):e0329878. doi: 10.1371/journal.pone.0329878 (PMC12517476; doi:10.1371/journal.pone.0329878)
Supplement: S3 Table — (PDF) [file pone.0329878.s003.pdf]

S3 Table. JMTV recombination analysis sequences.

Sample collection site	Year of collection	Tick Species/Host	Segment 1	Segment 2	Segment 3	Segment 4
**Isiolo, Kenya**	2023	*Rhipicephalus boophilus microplus*	PV384487(3079bp)	PV384450(2845bp)	PV384482(2812bp)	PV384509(2758bp)
**Isiolo, Kenya**	2023	*Amblyomma lepidium*	PV384490(3047bp)	PV384453(2775bp)	PV384484(2747bp)	PV384512(2726bp)
**Lamu, Kenya**	2022	*Rhipicephalus boophilus microplus*	PV384491(3045bp)	PV384454(2776bp)	PV384485(2802bp)	PV384513(2814bp)
**Uganda**	2012	*Piliocolobus rufomitratus*	KX377513(2950bp)	KX377514(2326)	KX377515(1996bp)	KX377516(2741)
**China**	2010	*Rhipicephalus boophilus microplus*	NC024113(3114bp)	NC024112(2847bp)	NC024114(2824bp)	NC024111(2794bp)
**Kenya**	2019	*Amblyomma spp*	ON186499(2970bp)	ON186506(2543bp)	ON186513(2637bp)	ON186520(2502bp)
